# Enhanced expression of ADCY1 underlies aberrant neuronal signalling and behaviour in a syndromic autism model

**DOI:** 10.1038/ncomms14359

**Published:** 2017-02-20

**Authors:** Ferzin Sethna, Wei Feng, Qi Ding, Alfred J. Robison, Yue Feng, Hongbing Wang

**Affiliations:** 1 Genetics Program, Michigan State University, East Lansing, Michigan 48824, USA; 2 Department of Pharmacology, Emory University School of Medicine, Atlanta, Georgia 30322, USA; 3 Department of Physiology, Michigan State University, East Lansing, Michigan 48824, USA; 4 Neuroscience Program, Michigan State University, East Lansing, Michigan 48824, USA

## Abstract

Fragile X syndrome (FXS), caused by the loss of functional FMRP, is a leading cause of autism. Neurons lacking FMRP show aberrant mRNA translation and intracellular signalling. Here, we identify that, in *Fmr1* knockout neurons, type 1 adenylyl cyclase (*Adcy1*) mRNA translation is enhanced, leading to excessive production of ADCY1 protein and insensitivity to neuronal stimulation. Genetic reduction of *Adcy1* normalizes the aberrant ERK1/2- and PI3K-mediated signalling, attenuates excessive protein synthesis and corrects dendritic spine abnormality in *Fmr1* knockout mice. Genetic reduction of *Adcy1* also ameliorates autism-related symptoms including repetitive behaviour, defective social interaction and audiogenic seizures. Moreover, peripheral administration of NB001, an experimental compound that preferentially suppresses ADCY1 activity over other ADCY subtypes, attenuates the behavioural abnormalities in *Fmr1* knockout mice. These results demonstrate a connection between the elevated *Adcy1* translation and abnormal ERK1/2 signalling and behavioural symptoms in FXS.

## Abstract (web summary)

Fragile X syndrome (FXS) is a leading cause of autism and neurons lacking FMRP show aberrant mRNA translation and intracellular signalling. Here, the authors show that neurons from Fmr1 knockout mice have increased levels of ADCY1 protein, producing abnormal ERK1/2 signalling, dysregulated protein synthesis and behavioural symptoms associated with FXS.

Loss of the functional fragile X mental retardation protein (FMRP) encoded by the *FMR1* (Fragile X mental retardation 1) gene^1^ is responsible for the cellular and behavioural abnormalities in Fragile X syndrome (FXS)^2^,^3^. In addition to intellectual disability, FXS patients often express autism-related symptoms, including repetitive behaviour and impaired social interaction^3^,^4^,^5^. Increased dendritic spine density and immature spines are observed in FXS postmortem brains^6^. Many of the FXS phenotypes have been recapitulated in the *Fmr1* knockout (KO) mouse model, in which the *Fmr1* gene is deleted^3^,^7^.

Biochemical studies have demonstrated that FMRP interacts with specific mRNAs and is associated with translating polyribosomes to regulate translation of these target mRNAs in the brain^2^,^8^,^9^. It is estimated that FMRP directly interacts with 800 to 6,000 different mRNA targets^10^,^11^,^12^. The loss of functional FMRP results in aberrantly increased basal level translation of FMRP target mRNAs in FXS patient cells and in the mouse model of FXS^13^,^14^. Another molecular abnormality found in both human and mouse FXS samples is enhanced signal transduction in the ERK1/2 (extracellular signal-regulated kinases 1 and 2) and PI3K (phosphoinositide 3-kinase) pathways^15^,^16^,^17^,^18^,^19^, which also lead to aberrantly enhanced protein translation through activating S6K1 (ribosomal protein S6 kinase beta-1)^20^,^21^. The dendritic spine abnormalities in *Fmr1* deficient neurons are thought to be due to the lack of activity-dependent translational regulation at synapses^22^,^23^. Although mRNA encoding the p110β subunit of PI3K is a direct target of FMRP, which may explain the deregulation of PI3K signalling in FSX^15^,^24^, how the loss of FMRP-dependent translation regulation leads to hyperactivity of ERK1/2 signalling is not understood. Moreover, whether translational dysregulation of specific FMRP target mRNA(s) is causal for autism-related behavioural symptoms in FXS remains elusive.

Type 1 adenylyl cyclase (ADCY1) is a neurospecific protein that catalyses cAMP production and is preferentially enriched at the postsynaptic density^25^,^26^. As ADCY1 activity can be dynamically regulated by calcium and neuronal stimulation, its function has been implicated in regulating neuronal signal transduction and synaptic plasticity^27^. Overexpression of *Adcy1* in mouse forebrain causes enhanced ERK1/2 activation^28^ and reduced sociability^29^, recapitulating some molecular and autism-related phenotypes in *Fmr1* KO mouse. Interestingly, previous high-throughput screening studies identified interaction of FMRP with the *Adcy1* mRNA^10^,^11^,^12^. Here, we find that *Adcy1* mRNA translation is aberrantly increased in the absence of FMRP and altered ADCY1 expression contributes to the enhanced ERK1/2 signalling and autism-related behaviours in *Fmr1* KO mice.

## Results

### FMRP suppresses *Adcy1* mRNA translation

**Figure 1:**
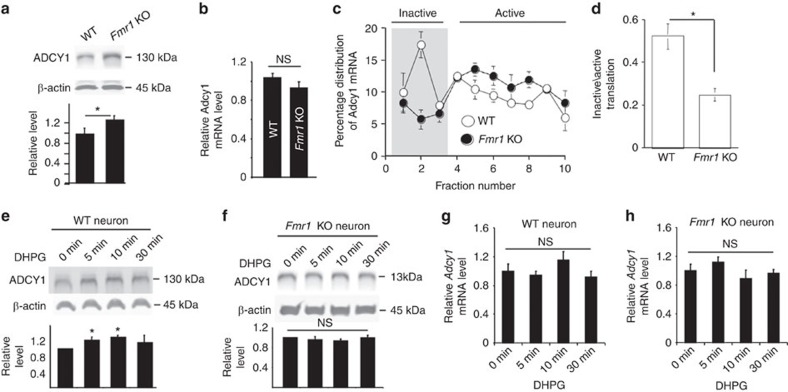
FMRP negatively regulates *Adcy1* mRNA translation. (**a**) The level of ADCY1 protein from the hippocampus of WT and *Fmr1* KO mice. The representative Western blot result is shown in the top panel, and quantification (normalized to β-actin) is shown in the bottom panel. *P=0.021, *n*=6 per group, Student's *t*-test. (**b**) *Adcy1* mRNA level in WT and *Fmr1* KO hippocampus was determined by qRT-PCR (*n*=6 per group). (**c**) Distribution of *Adcy1* mRNA in different ribosome fractions obtained by linear sucrose gradient centrifugation from hippocampal lysates of WT (*n*=3) and *Fmr1* KO mice (*n*=3). (**d**) The ratio of *Adcy1* mRNA in ribosome fractions 1–3 to fractions 4–10. *P=0.026, *n*=3 per group, Student's *t*-test. (**e**–**h**) Cultured WT (**e**,**g**) and *Fmr1* KO (**f**,**h**) hippocampal neurons (*n*=6 per group) were stimulated with the mGluR1/5 agonist DHPG (100 μM) for various durations (as indicated). The level of ADCY1 protein (**e**,**f**) and mRNA (**g**,**h**) following DHPG treatment were determined by western blot (normalized to β-actin) and qRT-PCR (normalized to *Gapdh* mRNA level), respectively. *: significant difference between control and the indicated group; *P*=0.032 (for the 5 min post-treatment group) and *P*=0.046 (for the 10 min post-treatment group), determined by one-way ANOVA (*n*=6 for each group, *F*_3,20_=3.927, *P*=0.021) followed by LSD *post-hoc* analysis (**e**). NS: not significant. Data are presented as mean±s.e.m.

By using an ADCY1-specific antibody (Supplementary Fig. 1), we found that the level of ADCY1 protein was significantly increased (about 25%) in the hippocampus of *Fmr1* KO mice as compared with the wild type (WT) controls (Fig. 1a). In contrast, *Adcy1* mRNA levels were not affected by the loss of FMRP (Fig. 1b), suggesting that FMRP regulates *Adcy1* mRNA translation. To directly test this hypothesis, we performed linear sucrose gradient fractionation to assess polyribosome association of the *Adcy1* mRNA^30^. In WT hippocampus, a significant fraction of *Adcy1* mRNA (∼34.5%) was sequestered into translational quiescent messenger ribonucleoprotein (mRNP) complexes (Fractions 1–3, Fig. 1c,d), and ∼65.5% of *Adcy1* mRNA was engaged with translating polyribosomes (Fractions 4–10, Fig. 1c,d). In the *Fmr1* KO hippocampus, less *Adcy1* mRNA (∼20.5%) was detected in the inactive mRNPs, whereas a reciprocal increase of polyribosome association with *Adcy1* mRNA was observed (∼79.5%) (Fig. 1c,d). These data indicate that FMRP suppresses *Adcy1* translation in resting hippocampus.

Because mGluR1/5-mediated activity-dependent translation of postsynaptic proteins requires FMRP^23^,^31^,^32^,^33^,^34^, we applied the mGluR1/5 agonist DHPG, which activates the downstream intracellular signalling target ERK1/2 in both WT and *Fmr1* KO neurons (Supplementary Fig. 2a,b). However, while mGluR1/5 activation caused up-regulation of ADCY1 protein in WT neurons (Fig. 1e), there was no DHPG-triggered increase of ADCY1 protein in *Fmr1* KO neurons (Fig. 1f). The activation of mGluR1/5 did not alter *Adcy1* mRNA levels (Fig. 1g,h). Altogether, our data demonstrate that FMRP regulates *Adcy1* mRNA translation under basal as well as stimulated conditions.

### Excessive ADCY1 contributes to aberrant signalling in FXS

**Figure 2:**
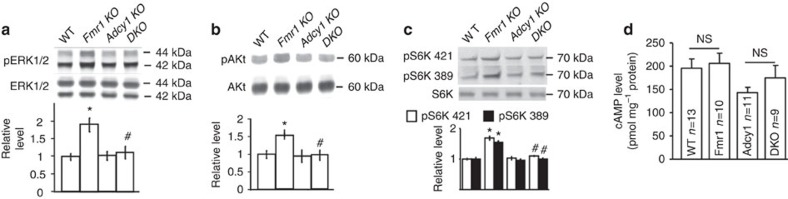
Genetic removal of *Adcy1* normalizes aberrant neuronal signalling in *Fmr1* KO hippocampus. The levels of phosphorylated and total ERK1/2 (**a**), Akt (**b**) and S6K1 (**c**) in the hippocampus of WT, *Fmr1* KO, *Adcy1* KO and DKO mice (*n*=6 per group) were determined by immunoblot. Representative immunoblot results are shown in the top panels, and quantifications are shown in the bottom panels. (**d**) The level of cAMP in the hippocampus of WT, *Fmr1* KO, *Adcy1* KO and DKO mice was determined by ELISA. *: significant difference between WT and *Fmr1* KO group (*P*=0.021 in a; *P*=0.033 in **b**; *P*=0.037 and 0.036 for pS6K421 and pS6K389, respectively, in **c**). ^#^: significant difference between *Fmr1* KO and DKO group (*P*=0.028 in **a**; *P*=0.026 in **b**; *P*=0.033 and 0.031 for pS6K421 and pS6K389, respectively, in **c**). The *P*-values were determined by *post-hoc* LSD test following ANOVA analysis (*n*=6 for each group; *F*_3,20_=5.354, *P*=0.009 in **a**; *F*_3,20_=4.662, *P*=0.02 in **b**; *F*_3,20_=3.525, *P*=0.029 for pS6K421 and *F*_3,20_=3.747, *P*=0.027 for pS6K389 in **c**). NS: not significant. Data are presented as mean±s.e.m.

As previous studies indicated that transgenic overexpression of ADCY1 leads to enhanced ERK1/2 activation^28^, we hypothesize that aberrant ADCY1 expression in *Fmr1* KO mice is causal for the elevated ERK1/2-mediated signalling. To test this hypothesis, we generated double KO (DKO) mice in which both *Fmr1* and *Adcy1* were genetically deleted. Indeed, we detected increased activity of both ERK1/2 (as indicated by the higher level of p-ERK1/2, Fig. 2a) and PI3K (as indicated by the higher level of pAkt) (Fig. 2b) in *Fmr1* KO hippocampus^15^,^16^,^17^,^18^, which resulted in hyper-phosphorylation of their downstream target S6K1 (Fig. 2c)^20^. Importantly, genetic reduction of *Adcy1* normalized the elevated level of p-ERK1/2, pAkt and pS6K1 (at the ERK1/2 target site Thr421/Ser424 and the PI3K target site Thr389) (Fig. 2a–c) in DKO mice, suggesting that the loss of FMRP-dependent translation suppression of *Adcy1* is a prevailing cause for aberrant neuronal signalling in FXS. Interestingly, we found that the total cAMP level is less responsive to the elevated ADCY1 expression in *Fmr1* KO hippocampus. Total level of cAMP in hippocampus was not significantly different between WT and *Fmr1* KO mice (Fig. 2d). *Adcy1* KO hippocampus displayed a significant reduction of cAMP, the level of which is comparable to that in DKO hippocampus (Fig. 2d). Nonetheless, the correction of ERK1/2–S6K1 and PI3K–S6K signalling in DKO hippocampus suggests that the aforementioned signalling pathways are causally coupled to ADCY1 overproduction in *Fmr1* KO neurons.

### Genetic reduction of *Adcy1* corrects abnormalities in FXS

**Figure 3:**
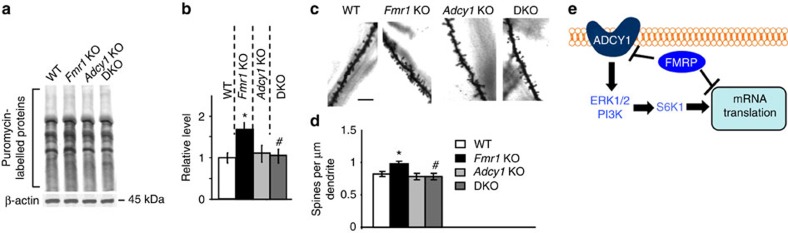
Genetic removal of *Adcy1* normalizes cellular abnormalities in *Fmr1* KO mice. (**a**) Newly synthesized proteins in primary hippocampal neurons from WT, *Fmr1* KO, *Adcy1* KO and DKO mice were labelled by puromycin, and detected by anti-puromycin antibody. (**b**) The intensity of protein bands between 15 and 250 kDa was quantified and normalized to the level of β-actin. (**c**) Golgi staining of dendritic spines on apical dendrites in the CA1 area of WT, *Fmr1* KO, *Adcy1* KO and DKO mice (length of the scale bar, 10 μm). (**d**) Quantification of total spine number on the CA1 apical dendrites. (**e**) A working model illustrates the function of FMRP-dependent suppression of Adcy1 translation in controlling intracellular signalling and neuronal protein synthesis. *: significant difference between WT and *Fmr1* KO group (*P*=0.018 in **b**; *P*=0.025 in **d**). ^#^: significant difference between *Fmr1* KO and DKO group (*P*=0.041 in **b**; *P*=0.038 in **d**). The *P*-values were determined by *post-hoc* LSD test following ANOVA analysis (*n*=6 for each group, *F*_3,20_=4.175, *P*=0.019 in **b**; *n*=4 for each group, *F*_3,12_=3.827, *P*<0.03 in **d**). Data are presented as mean±s.e.m.

Phosphorylation of S6K1 by ERK1/2 and PI3K is known to promote translation, which contributes to excessive protein synthesis and higher dendritic spine density in FXS^15^,^20^,^35^. Thus, we next tested whether genetic reduction of *Adcy1* can ameliorate aberrant protein synthesis, which may underlie dendritic spine abnormalities in *Fmr1* KO hippocampal neurons^36^. Consistent with previous reports showing that the elevated ERK1/2, PI3K and S6K1 activity enhances translation in FXS^15^,^20^,^35^,^37^, we observed increased basal level protein synthesis in *Fmr1* KO hippocampal neurons (Fig. 3a,b). As reported in previous studies^13^,^24^,^37^, we confirmed that the total spine density in *Fmr1* KO hippocampus (Fig. 3c,d) and visual cortex (Supplementary Fig. 3) was abnormally increased comparing to WT mice. While *Adcy1* KO mice did not show alterations in protein synthesis or spine density, genetic removal of *Adcy1* in the *Fmr1* KO mouse attenuated the aberrantly increased protein synthesis and dendritic spine density (Fig. 3; Supplementary Fig. 3). Altogether, our data support a working model that loss of FMRP-dependent suppression of *Adcy1* mRNA translation in FXS results in exaggerated ERK1/2 signalling, which cross talks with PI3K and impinges on S6K1. Consequently, S6K1 is hyper-phosphorylated and may, in turn, lead to aberrantly increased protein synthesis in *Fmr1* KO neurons (Fig. 3e).

**Figure 4:**
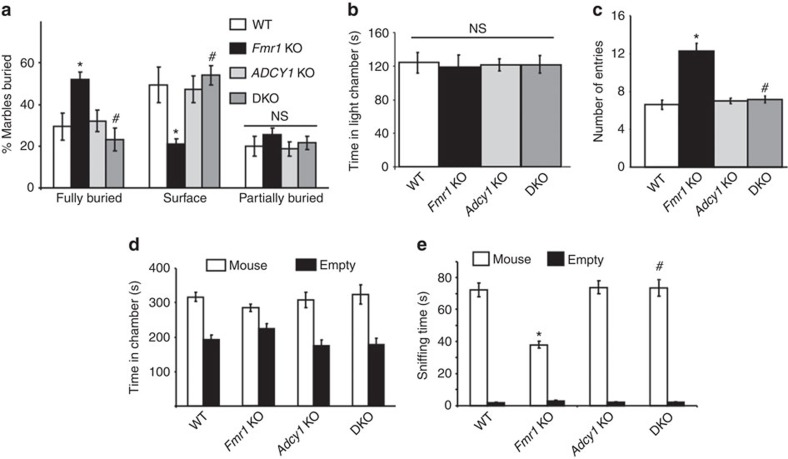
Genetic removal of *Adcy1*attenuates autism-related symptoms in *Fmr1* KO mice. (**a**) Marble burying test with WT (*n*=10), *Fmr1* KO (*n*=15), *Adcy1* KO (*n*=10) and DKO (*n*=17) mice. The % of fully buried, surface and partially buried marbles was recorded. (**b**,**c**) In the light/dark test, the time spent in the lit chamber (**b**) and the number of entries to the lit chamber (**c**) were recorded in WT (*n*=12), *Fmr1* KO (*n*=12), *Adcy1* KO (*n*=12) and DKO (*n*=9) mice. (**d**,**e**) The 3-chamber social interaction test was performed with WT (*n*=11), *Fmr1* KO (*n*=11), *Adcy1* KO (*n*=10) and DKO (*n*=10) mice. Time spent in the chamber having the stimulus mouse-containing enclosure or the empty enclosure was recorded (**d**). Direct social interaction between the test mouse and the stimulus mouse was determined by the amount of sniffing time during the social interaction test (**e**). *: significant difference between WT and *Fmr1* KO group (*P*=0.033 and *P*=0.017 for the fully buried and surface marbles, respectively, in **a**; *P*=0.02 in **c**; *P*=0.021 in **e**). ^#^: significant difference between *Fmr1* KO and DKO group (*P*=0.024 and *P*=0.013 for the fully buried and surface marbles, respectively, in **a**; *P*=0.014 in **c**; *P*=0.019 in **e**). The *P*-values were determined by *post-hoc* LSD test following ANOVA analysis (*F*_3,48_=3.255, *P*=0.037 for fully buried marbles in **a**; ANOVA *F*_3,48_=3.282, *P*=0.036 for surface marbles in **a**; *F*_3,41_=3.31, *P*=0.033 in **c**; *F*_3,38_=3.054, *P*=0.031 in **e**). NS: not significant. Data are presented as mean±s.e.m.

To further test the functional relevance of elevated ADCY1 expression in FXS, we examined whether genetic removal of *Adcy1* can correct behavioural symptoms in *Fmr1* KO mice. Recapitulating the autistic phenotypes in FXS patients, *Fmr1* KO mice showed repetitive behaviour and reduced social interaction. In the marble burying test, *Fmr1* KO mice buried more marbles than WT and *Adcy1* KO mice (Fig. 4a). In the light/dark test, *Fmr1* KO mice spent normal time in the light chamber (Fig. 4b) but made more transitions between the light and dark chambers (Fig. 4c), indicating repetitive and hyperactive behaviour. In the 3-chamber social interaction test, all groups showed comparable time spent in the chamber that held the stimulus mouse (Fig. 4d). However, direct interaction between the *Fmr1* KO mice and the stimulus mouse was significantly reduced (Fig. 4e). Both repetitive behaviour and impaired social interaction were corrected in DKO mice (Fig. 4a–e). In contrast, genetic deletion of *Adcy1* alone (in *Adcy1* KO mice) did not alter these autism-related behaviours (Fig. 4a–e).

Consistent with the higher seizure susceptibility in autism and FXS patients, *Fmr1* KO mice showed audiogenic seizures (AGS), which were absent in WT and *Adcy1* KO mice (Table 1). AGS was significantly reduced in DKO mice (Table 1). As homozygous *Adcy1* mutation in human is linked to mild-to-moderate mixed hearing impairment^38^, we examined whether attenuation of AGS in DKO mice is due to insensitivity to acoustic stimulation. Interestingly, *Fmr1* KO, *Adcy1* KO and *Adcy1/Fmr1* DKO mice showed higher startle response than WT animals (Supplementary Fig. 4). In addition, *Adcy1* KO mice show normal tone-associated learning and memory^39^, indicating no severe hearing deficits. Together, these results demonstrate that genetic deletion of *Adcy1* in *Fmr1* KO mouse rescues several well-characterized autism-related symptoms.

### Acute inhibition of ADCY1 corrects abnormalities in FXS

**Figure 5:**
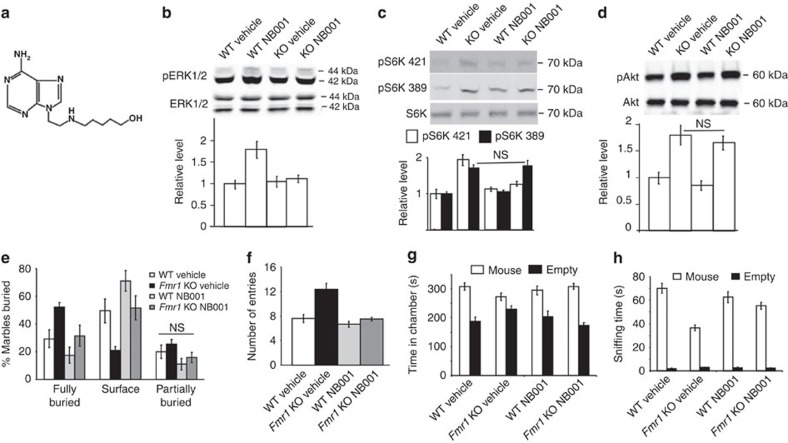
Acute pharmacological inhibition of ADCY1 rescues the abnormal ERK1/2 activity and autism-related symptoms in *Frm1* KO mice. (**a**) Molecular structure of NB001. (**b**–**h**) WT and *Fmr1* KO mice received i.p. injection of NB001 (1 mg kg^−1^) or vehicle. Biochemical or behavioural examinations were performed one hour after injection. (**b**–**d**) The levels of phosphorylated and total level of ERK1/2 (**b**), S6K1 (**c**) and Akt (**d**) in the hippocampus of WT and *Fmr1* KO mice receiving vehicle or NB001 (*n*=5 for each group) were determined by immunoblot. Representative immunoblot results are shown in the top panels, and quantifications are shown in the bottom panels. (**e**) Mice receiving vehicle (WT, *n*=9; *Fmr1* KO, *n*=12) or NB001 (WT, *n*=9; *Fmr1* KO, *n*=9) were examined by the marble burying test. (**f**) In light/dark test (WT vehicle *n*=11, *Fmr1* KO vehicle *n*=13, WT NB001 *n*=10, *Fmr1* KO NB001 *n*=15), number of entries to the lit chamber was recorded. (**g**,**h**) In the 3-chamber social interaction test (WT vehicle *n*=12, *Fmr1* KO vehicle *n*=12, WT NB001 *n*=10, *Fmr1* KO NB001 *n*=12), time spent in the chamber having the stimulus mouse-containing enclosure or the empty enclosure was recorded (**g**). Direct social interaction between the test mouse and the stimulus mouse was determined by the amount of sniffing time during the social interaction test (**h**). Detected by two-way ANOVA, NB001 corrected the elevated pERK (*F*_1,16_=5.432, *P*=0.033 in **b**) and pS6K421 (*F*_1,16_=5.833, *P*=0.028 in **c**), abnormal marble burying (*F*_1,35_=4.124, *P*=0.041 for fully buried marbles; *F*_1,35_=4.12, *P*=0.041 for surface marbles in **e**), light/dark behaviour (*F*_1,45_=4.611, *P*=0.029 in **f**) and abnormal direct social interaction (*F*_1,42_=5.975, *P*=0.031 in **h**) in *Fmr1* KO mice. NS: not significant. Data are presented as mean±s.e.m.

We took advantage of a newly discovered small compound NB001 (5-{[2-(6-amino-9H-purin-9-yl)ethyl]amino}-1-pentanol) (Fig. 5a), which preferentially inhibits ADCY1 activity over other types of adenylyl cyclases in intact cells^40^. Mouse liver microsome assay demonstrated that NB001 was stable and resistant to degradation (Supplementary Fig. 5). Following intraperitoneal (IP) injection, NB001 was absorbed to the blood stream (Supplementary Fig. 6a) and distributed to the brain (Supplementary Fig. 6b). These data, together with the report showing that systemic administration of NB001 attenuates neurophathic pain^40^, demonstrate that NB001 can cross the blood–brain barrier.

Previous study used 1 mg kg^−1^ NB001 (through IP injection) to attenuate ADCY1 activity and neuropathic pain^40^. We used the same dose and found that, following NB001 administration, the elevated levels of p-ERK1/2 and pS6K1 (at Thr421/Ser424) were normalized in the hippocampus of *Fmr1* KO mice to the WT levels (Fig. 5b,c). The levels of pS6K1 at PI3K target site Thr389 (Fig. 5c) and pAkt (Fig. 5d) were not affected by acute administration of NB001.

The NB001-injected *Fmr1* KO and WT mice showed comparable marble burying behaviour (Fig. 5e) and transition between the light and dark chambers (Fig. 5f and Supplementary Fig. 7). NB001 also normalized the reduced social interaction in *Fmr1* KO mice to the WT level (Fig. 5g,h). Furthermore, *Fmr1* KO mice receiving NB001 showed less occurrence of AGS when compared with the vehicle (saline)-injected controls (Table 2). Importantly, NB001 used in these experiments did not cause measurable changes in the phosphorylation of ERK1/2 and S6K1 (Fig. 5b–d) as well as behaviour in WT mice (Fig. 5e–h; Table 2). These results demonstrate that acute pharmacological inhibition of ADCY1 activity is effective in correcting the abnormal ERK1/2 activity and autism-related symptoms in the FXS mouse model.

**Figure 6:**
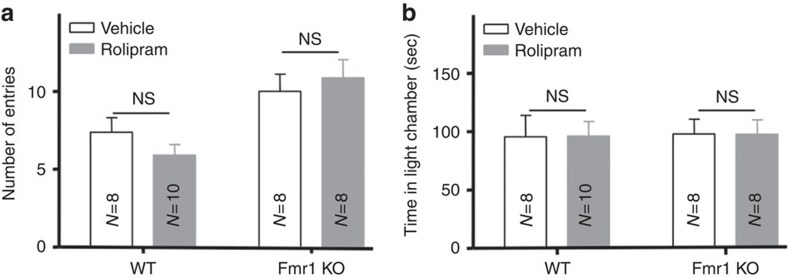
Acute pharmacological inhibition of PDE has no significant effect on the FXS-associated phenotype in light/dark test. WT and *Fmr1* KO mice received i.p. injection of rolipram (0.5 mg kg^−1^) or vehicle. Thirty minutes after injection, mice were examined by light/dark test as described in Fig. 4. Regardless of treatment, *Fmr1* KO mice showed more transition between the lit and dark chamber than WT mice (genotype effect: F_1,30_=15.2, *P*=0.001. two-way ANOVA) (**a**), and normal time in the lit chamber (**b**). Rolipram does not cause significant behavioural changes in either WT or *Fmr1* KO mice (drug effect: F_1,30_=0.095, *P*=0.76, two-way ANOVA). NS: not significant. The numbers of animals for each group are indicated in the figure. Data are presented as mean±s.e.m.

Previous studies reported reduction of cAMP level in the drosophila model of FXS (ref. 41 but also see ref. 42) and that increasing cAMP by administration of the phosphodiesterase (PDE) inhibitor rolipram has therapeutic effects on correcting learning and memory deficits in FXS flies^43^. Here, we assessed whether the aforementioned autism-associated behaviour abnormalities in the *Fmr1* KO mice can be attenuated by rolipram. We injected rolipram at 0.5 or 0.03 mg kg^−1^ representing medium/high and low dose, respectively, according to previous studies to inhibit PDE^41^,^44^,^45^. Systemic administration of rolipram at 0.5 or 0.03 mg kg^−1^ did not correct the abnormal behaviour in light/dark test (Fig. 6; Supplementary Fig. 8) and AGS (Table 3; Supplementary Table 1). The results suggest that these behavioural symptoms in *Fmr1* KO mice are sensitive to ADCY1 rather than PDE inhibition.

Since drug toxicity is a major concern in therapeutic development, we examined the effects of NB001 on some health-related parameters. Mice were injected with NB001 at 50 mg kg^−1^, which is 50 times higher than the therapeutic dose, twice a day for 14 days. This treatment did not affect body weight (Supplementary Fig. 9a) as well as the weight of major organs including heart/lung, kidney and liver (Supplementary Fig. 9b). There was no significant sign of histopathology in kidney and liver (Supplementary Fig. 9c,d). The data from the clinical panel blood test showed that NB001 had no effect on concentrations of various electrolytes and levels of the main toxicity-related proteins (such as aminotransferases) (Supplementary Table 2). These results indicate no significant side effects following two weeks of high dose NB001 administration. The effects of high dose NB001 on other health-related domains including behavioural alteration may be investigated in the future studies.

## Discussion

**Figure 7:**
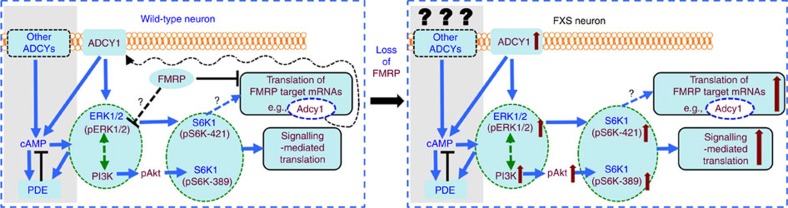
Function of ADCY1 in connecting FMRP-regulated translation and exaggerated ERK1/2–S6K1 signalling in FXS. Signalling molecules affected in FXS, translation of direct FMRP mRNA targets (including Adcy1), and translation under control of Adcy1–ERK1/2 signalling are marked in maroon font. The loss of FMRP-dependent suppression of *Adcy1* mRNA translation results in overexpression of ADCY1 on synaptic membrane, which leads to exaggerated ERK1/2 signalling (possibly cross talks with PI3K pathway), increased S6K1 activity and enhanced protein synthesis in FXS neurons. A possible feed forward loop, in which ADCY1-mediated ERK1/2 over-activation further enhances Adcy1 mRNA translation, may amplify exaggerated neuronal signalling and translation dysregulation in FXS. The homoeostasis of cAMP is maintained by the balance between multiple ADCYs and PDE, whose activity can be up-regulated by cAMP, ERK1/2 and PI3K. It is unclear whether the elevated ERK1/2 activity caused by ADCY1 overexpression is solely mediated by cAMP production. Insights about how FMRP-dependent translation impinges on the feedback network among these signalling molecules require further investigation.

Elevated neuronal signalling and enhanced basal protein synthesis have been proposed as the prevailing mechanisms underlying the pathophysiology of FXS^2^,^36^. However, whether and how these cellular abnormalities are connected is largely unknown. In this study, we identify the altered ADCY1 as a missing link connecting the FMRP-regulated translation and the aberrantly increased activity of the ERK1/2–S6K1 pathway and signalling-mediated increase of protein synthesis in *Fmr1* KO neurons (Fig. 7). Moreover, we demonstrate that hyperactivity of ADCY1-directed neuronal signalling is causative for autism-related core behavioural abnormalities in the *Fmr1* KO mouse model of FXS, which can be reversed by genetic and pharmacological reduction of ADCY1. Thus, the neuronal specific ADCY1–ERK1/2 signalling pathway revealed by our studies offers a potential target for developing therapeutic strategies against autism-related symptoms.

Exaggerated protein synthesis in FXS is caused by both the loss of translation suppression of direct FMRP ligand mRNAs^9^ and the increased neuronal signalling by ERK1/2 and PI3K through phosphorylation of S6K1^20^,^24^,^35^,^37^. Although FMRP binds the mRNA of S6K1, S6K1 protein expression is not elevated in FXS^17^,^21^. Identification of the PI3K p110ß subunit and PI3K activator PIKE as direct targets of FMRP may explain elevated PI3K signalling in FXS^17^,^24^,^37^, whereas how the loss of FMRP-dependent translation suppression leads to elevated ERK1/2 signalling in FXS has remained mysterious. Our study reveals *Adcy1* mRNA as a target for FMRP-dependent translation regulation in both resting and stimulated neurons. Notably, besides the enhanced basal level *Adcy1* mRNA translation, mGluR1/5-dependent increase of ADCY1 is lost in *Fmr1* KO neurons, recapitulating the lack of activity-dependent translation up-regulation in FXS^13^,^35^. Using genetic and pharmacological approaches, we further demonstrate that excessive production of ADCY1 protein in resting neurons lacking FMRP is a prevailing cause for the enhanced activity of ERK1/2 and S6K1, consistent with the fact that ADCY1 and cAMP signalling are positive regulators of ERK1/2 in neurons^28^,^46^,^47^. The functional importance of ADCY1 in FXS pathophysiology is further indicated by the fact that reduction of ADCY1 function concurrently ameliorates exaggerated protein synthesis and ERK1/2–S6K1 signalling in *Fmr1* KO neurons. Besides attenuating ERK1/2 signalling, long-term genetic reduction of *Adcy1* in *Fmr1* KO mice also dampened the elevated pAkt and pS6K1 at Thr389 (a PI3K target phosphorylation site), suggesting a novel cross talk between ADCY1 and PI3K. Interestingly, reduction of ADCY1 function does not suppress ERK1/2 and PI3K signalling and protein synthesis in WT mice, similar to the fact that reduction/inhibition of ERK1/2, PI3K and S6K1 specifically dampens protein synthesis in FXS but not in WT neurons^20^,^24^,^35^,^37^.

ERK1/2 is activated by mGluR1/5, the hyper function of which has been demonstrated in FXS and a handful of other autism model mice^33^,^48^,^49^,^50^. In addition, ERK1/2 activity is elevated in both *Fmr1* KO mice and the BTBR mouse model of autism^16^,^18^,^51^. The striking effects of genetic reduction of *Adcy1* on both ERK1/2 activity and autism-related behaviour in *Fmr1* KO mice support the hypothesis that exaggerated ERK1/2 signalling significantly contributes to the autism-associated symptoms, which encourages future investigation in other autism models that shares common abnormality in mGluR1/5 and ERK1/2. Notably, *Adcy1* KO mice display no autism-related behaviour, the therapeutic correction on behaviour by genetic and pharmacological manipulation of ADCY1 is specific when neuronal signalling is exaggerated.

Although ADCY1 expression is elevated in *Fmr1* KO neurons, we did not detect measurable difference of overall basal cAMP level between *Fmr1* KO and WT samples. Previous studies have also reported that the basal levels of cAMP do not differ in samples collected from human FXS or *Fmr1* KO mice^42^,^52^. One possibility is that there are 10 difference ADCYs, all contributing to cAMP production. The current cAMP assay may not be sufficiently sensitive to detect overall cAMP alteration caused by the 25% increase of ADCY1 expression in FXS. Another possibility is that lack of FMRP may also cause alterations in other ADCYs that are not directly coupled to ERK activation. Moreover, cAMP itself and the ADCY1-mediated elevation of ERK/PI3K activity may up-regulate certain phosphodiesterases (such as PDE4) (refs 53, 54, 55, but also see ref. 56), which may in turn, as a secondary pathological outcome, counter balance the elevated ADCY1 activity and maintain cAMP homoeostasis in FXS (Fig. 7). Development of more sensitive cAMP assays and better isolation of distinct ADCY and PDE function may help to determine the ADCY1 effects on cAMP level in FXS. Alternatively, the possibility of cAMP-independent function of ADCY1-mediated ERK1/2 and translation may be explored in the future. Interestingly, previous studies found that forskolin-stimulated cAMP production is ablated rather than decreased in FXS patient cells and *Fmr1* KO mouse brains^42^,^52^,^57^. Considering that forskolin is not endogenous and can affect all subtypes of ADCYs but ADCY9, the null rather than lower or higher response to forskolin in FXS cells is intriguing and requires further investigation. Nonetheless, the success of rescuing neuronal signalling and behavioural abnormalities in *Fmr1* KO mice by genetic and pharmacological reduction of ADCY1 function suggests that ADCY1-coupled signalling is a major factor for pathophysiology in the mouse model of FXS.

Previous studies have suggested an alternative mechanism in the fly model of FXS regarding how FMRP may affect cAMP signalling. Two recent studies observed lower basal cAMP levels in the whole head of FXS fly^41^,^58^, although no reduction of cAMP was observed in FXS flies in an earlier report^42^. Notably, enhancement of cAMP by the PDE inhibitor rolipram improved learning and memory in the fly model of FXS^41^. In *Fmr1* KO mice, rolipram treatment rescued the exaggerated mGluR1/5-mediated long-term synaptic depression phenotype^41^ but failed to correct the signalling abnormalities in the hippocampus^43^. How PDE inhibition affects hippocampus-specific behaviour remains un-determined. Here, we found that rolipram does not attenuate AGS and abnormal behaviour in light/dark test, which may involve many brain regions rather than being solely dependent on hippocampus function, in *Fmr1* KO mice. This is in contrast to the rescue of key Fragile X-associate symptoms achieved by pharmacological inhibition and genetic reduction of *Adcy1*. It is critical to note that cAMP and cAMP-mediated signalling are positive regulators of ERK1/2 in mammalian neurons^46^,^47^. Transgenic overexpression of ADCY1 in mice, which is positively coupled to cAMP signalling in brain neurons, leads to elevated p-ERK1/2 level and reduced social interaction^28^,^29^, both of which are evident in *Fmr1* KO mice. Moreover, the enhanced ADCY1 expression caused by the loss of FMRP is consistent with multiple reports showing that the activity of ERK1/2 and its downstream target S6K1 is elevated in both mouse model and human FXS^16^,^17^,^18^,^20^. These observations open a potential future direction to investigate whether cAMP and particularly the balance between ADCY and PDE are uniquely regulated in distinct animal models.

Because Adcy1 expression is restricted in the central nervous system and under tight regulation^26^,^27^, it offers a unique opportunity of therapeutic development to treat neurological disorders without affecting the periphery. Although autism and FXS are developmental disorders, acute pharmacological inhibition of ADCY1 by NB001 effectively corrects the autism-related symptoms in adult *Fmr1* KO mice, suggesting that ADCY1 may be a promising target in treating autism-related symptoms even after the critical developmental window has passed.

Recent clinical trials have indicated significant challenges in the effort to translate therapeutic strategies from mouse studies to human application^59^,^60^. It is recognized that, while mGluR5 antagonism has robust efficacy in *Fmr1* KO mice^13^,^16^,^61^, similar pharmacological intervention only shows mild effects in a subpopulation of human FXS patients^60^. In addition to the common issues including clinical assessment, tolerance and gene–drug interaction, the less than expected therapeutic outcome may be due the existence of other pathological factors. Closely related to mGluR1/5, another group of Gq-coupled muscarinic acetylcholine receptors is also hyperactivated in *Fmr1* KO brain^62^,^63^. Considering that the ERK1/2–S6K1 and PI3K–S6K1 signalling cascades commonly mediate multiple Gq-coupled receptors and ADCY1 positively regulates both ERK1/2 and PI3K in FXS, inhibition of ADCY1 may offer a potential strategy to normalize the hyperactivity of multiple pathological factors (Fig. 7).

## Methods

### Animals

*Fmr1* KO mice were obtained from Dr Cara Westmark at University of Wisconsin Madison. *Adcy1* KO mice were previously reported^64^. Both *Fmr*1 KO and *Adcy1* KO mice^64^ have been bred into the C57BL6 background for more than 10 generations, and were used to obtain heterozygous double KO mice. By breeding *Fmr1*-/y *Adcy1*+/− (male) with *Fmr1*+/− *Adcy1*+/− (female), we obtained littermates of different genotypes and used these for both molecular and behavioural assays. Animals were housed in the university laboratory animal research facility and all the manipulations were in compliance with the guidelines of Institutional Animal Care and Use Committee at Michigan State University. The mice had *ad libitum* access to water and food and were housed in a 12 h dark/light condition. Male mice were used for experiments.

### Biochemical analyses

Hippocampal tissues were collected from 3-month-(+/−10 days) old mice. Inactive and active ribosomal complex were separated by sucrose gradient as described^30^, and the level of *Adcy1* mRNA in each fraction was determined by quantitative RT-PCR. The primers used for *Adcy1* were aaacacagtcaatgtggccagtcg (forward) and actttgcctctgcacacaaactgg (reverse). The protein levels in hippocampal tissues collected from mice were determined by western blot using anti-ADCY1 (Sigma, Cat # SAB4500146-100UG, 1:1,000 dilution), anti-p-ERK1/2 (Cell Signaling, Cat # 9101L, 1:1,000 dilution), anti-pAkt (Cell Signaling, Cat # 9271, 1:1,000 dilution), anti-pS6K1 (Cell Signaling, Cat # 9204L for the detection of phosphorylation at Thr421/Ser424, Cat # 9234S for the detection of phosphorylation at Thr389, 1:1,000 dilution), anti-ERK1/2 (Cell Signaling, Cat # 9102L, 1:1,000 dilution), anti-Akt (Cell Signaling, Cat # 9272S, 1:1,000 dilution), anti-S6K1 (Cell Signaling, Cat # 9202L, 1:1,000 dilution) and anti-β-actin antibody (Sigma, Cat # A5441; 1:5,000 dilution). Following incubations with infrared fluorescence (IRDye)-conjugated secondary antibodies (1:5,000, LI-COR Biosciences, Cat # 926–32211 and 926–68070), signals were detected using the Odyssey system (LI-COR Biosciences). The un-cropped western blots are shown in Supplementary Fig. 10.

Primary hippocampal neurons were cultured as described^65^, and used for the examination of mGluR1/5-mediated signalling and protein synthesis. Neurons were treated with 100 μM DHPG ((*RS*)-3,5-dihydroxyphenylglycine). Protein level and mRNA level were examined by western blot (for ADCY1, β-actin, p-ERK1/2 and ERK1/2) and quantitative RT-PCR (for *Adcy1* and *Gapdh* mRNA), respectively.

Protein synthesis was determined by the SUnSET method^20^,^66^. Hippocampal neurons were treated with 5 μg ml^−1^ puromycin (Sigma, Cat # P8833) for 30 min. Cell lysates were processed for Western blot using anti-puromycin antibody (KeraFAST, Cat # EQ0001, 1:1,000). The level of β-actin was used to determine total protein loading. ImageJ was used to measure the combined signal intensity of proteins with molecular weights ranging from 15 to 250 kDa.

### Dendritic spine analysis

Three-month-(+/−10 days) old mouse brains were processed according to manufacture instructions using the Fd Rapid GolgiStain Kit (FD NeuroTechnologies. Cat # PK401). Overall, 150-μm thick sections were cut using a vibratome, and images were collected using the × 100 objective on an Olympus FluoView 1,000 microscope. Spines on apical dendrites localized 50 to 100 μm from the cell bodies of hippocampal CA1 and primary visual cortex pyramidal neurons were counted using the NeuronStudio Version 0.9.92 software.

### cAMP assay

Hippocampus was dissected from 3-month-(+/−10 days) old mice and homogenized in 500 μl 0.1N HCl. The homogenate was centrifuged at 4 °C for 10 min and 10 μl of the supernatant was used in the assay. The cAMP level, expressed as pmole mg^−1^ protein, was determined by the cAMP complete ELISA kit (Enzo Life Sciences) according to the manufacture's protocol. The protein concentration was determined by Bradford assay.

### Behavioural testing

Repetitive behaviour was determined by the marble burying test as described^67^. 3-month-(+/−10 days) old mouse was placed in a regular mouse cage filled with 7.6 cm-deep bedding for 1 h before the test. The mouse was then briefly removed from the testing box and 15 marbles were evenly arranged in a 5 × 3 pattern on the surface of the bedding. The mouse was reintroduced into the testing box and was allowed to bury marbles for 10 min. At the end of the testing period, the number of marbles that were fully buried, partially buried and left on the surface was counted.

Light/dark test was examined as described in our previous study^7^. Light/dark test was performed using 3-month (+/−10 days) old mouse, which was first placed in the dark half of the chamber (Coulbourn Instruments). After 1 min of habituation, the trap door was opened and the mouse was allowed to move freely between the two chambers for 5 min. The time spent in each chamber and the number of entries into the lit side were recorded.

Audiogenic seizures (AGS) were examined with 21- to 24-day-old-mouse (7), as this phenotype is dependent on the age of the mouse and only detected in juvenile *Frm1* KO mouse. Mouse was placed in a box (30 cm *L* × 17 cm *W* × 12 cm *H*) with a flat plastic lid. A personal alarm (Streetwise, item # SWPDAL) was taped to the lid of the box and wired to a DC power supply to keep the sound amplitude constant. The mouse was allowed to acclimatize to the box for 5 min, following which 120 dB sound was emitted from the alarm for 2 min. The number of mice undergoing seizure activity within the 2-min period was counted. Audiogenic seizures were classified into different stages: wild running, clonic/tonic seizure and death.

Acoustic startle response was used to determine whether alteration in AGS is due to the difference in hearing-related function. As described in our previous study^7^, mouse (21- to 24-day old) was placed in the startle chamber of the SR-LAB apparatus (San Diego Instruments). Five trials of 120 dB startle stimulation were delivered randomly during the 5-min examination. Average startle response to the 5 stimulations was calculated and used for each mouse.

Social interaction was determined by the 3-chamber test as described^68^. Mouse at 3 months (+/−10 days) of age was placed in a 3-chamber social interaction box and allowed to explore freely for 5 min. If a mouse displayed a strong preference for a particular chamber during this time, it was omitted from the study. During the test, mouse was placed in the center chamber and the entrances to the side chambers were blocked. A novel stimulus mouse in a wire enclosure and an empty wire enclosure were placed in one of the side chambers, respectively. The entrances were then opened and the activity of the test mouse was recorded. Time spent in each of the chambers and time spent sniffing the stranger mouse enclosure versus the empty enclosure were recorded.

### Drug treatments

NB001 was dissolved in water to give a 25 mM stock solution. The stock was diluted in saline and IP injected into mice at 1 mg kg^−1^ body weight. In all cases, the drug was administered 1 h before testing. Control mice were treated similarly but injected with saline.

To inhibit phosphodiesterase (PDE), rolipram was dissolved in 10% kolliphore and IP injected into mice at 0.5 or 0.03 mg kg^−1^. The use of the higher dose (that is, 0.5 mg kg^−1^) was to ensure sufficient inhibition of PDE^44^,^45^. The lower dose (that is, 0.03 mg kg^−1^) was not sufficient to cause cAMP changes^69^ but was chosen, because the same dose was used to treat *Fmr1* KO mice and attenuated abnormality in synaptic depression^41^. Rolipram- or vehicle (10% kolliphore)-injected mice were examined with light/dark test and AGS 30 min after drug administration.

### NB001 toxicology studies

Mice at 3 months (+/−10 days) of age were IP injected with NB001 twice daily at 50 mg kg^−1^ for 2 weeks. Clinical observations were performed twice daily and body weights were measured on various days during the dosing regimen. At the end of the dosing period, mice were killed and blood was collected for clinical pathology (Diagnostic Center for Population and Animal Health, Michigan State University). The liver and kidney were collected and fixed in 10% buffered formalin (INVIVO facility at Michigan State University). Tissue morphology was revealed by H&E staining.

### NB001 pharmacokinetic studies

Following IP injection of NB001 (20 mg kg^−1^), mice were killed at various time points, and 0.5–0.8 ml of blood was drawn from a cardiac puncture into a 1 ml syringe, which was pre-treated with NaHeparin. The blood was collected into 1.5 ml microfuge tubes coated with NaHeparin and put on ice immediately. Samples were centrifuged at 15,000 r.p.m. for 15 min. Blood plasma was collected from the upper layer, leaving the blood cells in the tube. The plasma was frozen at −80 °C for later analysis. Brains were taken out and weighed immediately. Brains were frozen at −80 °C for later preparation and analysis. The samples were subjected to LC/MS/MS with Turbo-Ionspray Interface used in the positive ion-mode (Pharmacokinetics Core, University of Michigan). Mouse liver microsome stability assay was performed by the Pharmacokinetics Core at University of Michigan.

### Data collection and statistics

Experiments were performed with more than one mouse litter, from which mice used for distinct groups were equally and randomly divided. The number of repeat/sample was determined and based on previous studies and power analysis with pilot trials. For all data, normal distribution and equal variance were checked and followed by two-sided statistic tests. One-way ANOVA followed by *post-hoc* LSD (least significant difference) test or Student's *t*-test was used to compare multiple groups. Student's *t*-test was used to compare data from two groups. Two-way ANOVA followed by Student's *t*-test or LSD test was used to compare different groups in the behaviour studies and the SUnSET assay. χ^2^ test was used to analyse AGS data. Experimenters were blinded to the genotypes or treatments, which were decoded before data analysis. Data were expressed as mean±s.e.m. Differences with *P*-values <0.05 were considered significant. SPSS 11.5 for Windows was used for all data analysis.

### Data availability

All data supporting the findings of this study are available within the article and Supplementary Information files or from the corresponding author upon request.

## Additional information

**How to cite this article:** Sethna, F. *et al*. Enhanced expression of ADCY1 underlies aberrant neuronal signalling and behaviour in a syndromic autism model. *Nat. Commun.* **8,** 14359 doi: 10.1038/ncomms14359 (2017).

**Publisher's note**: Springer Nature remains neutral with regard to jurisdictional claims in published maps and institutional affiliations.

## Supplementary Material

Supplementary Information Supplementary Figures and Supplementary Tables

## Figures and Tables

**Table 1:** Genetic deletion of *Adcy1* attenuates audiogenic seizures.

**Genotype**	***N*** **(number)**	**% Wild running**	**% Clonic/tonic seizure**	**% Death**
WT	16	0	0	0
*Fmr1* KO	27	66.7	63	14.8
*Adcy1* KO	20	0	0	0
DKO	27	3.7	0	0

Audiogenic seizures were induced by sounds at the intensity of 120 dB. The percentage of different seizure-related phenotypes including wild running, clonic/tonic seizures, and death in WT, *Fmr1* KO, *Adcy1* KO and *Fmr1/Adcy1* double KO (DKO) is shown. χ^2^ test reveals that genetic deletion of *Adcy1* in *Fmr1* KO mice has significant correcting effect on the occurrence of audiogenic seizures (*P*=0.001 for wild running; *P*=0.001 for clonic/tonic seizure; *P*=0.038 for death). The numbers of animals are indicated in the table.

**Table 2:** Acute administration of NB001 attenuates audiogenic seizures.

**Genotype and treatment**	***N*** **(number)**	**% Wild running**	**% Clonic/tonic seizure**	**% Death**
WT+vehicle	15	0	0	0
*Fmr1* KO+vehicle	17	71.4	64.2	21.4
WT+NB001	16	0	0	0
*Fmr1* KO+NB001	17	35.2	5.8	5.8

Audiogenic seizures were induced by sounds at the intensity of 120 dB. The percentage of different seizure-related phenotypes including wild running, clonic/tonic seizures, and death in WT and *Fmr1* KO mice receiving vehicle or NB001 (1 mg kg^−1^) is shown. χ^2^ test reveals significant genotype effect (*P*=0.001 for wild running; *P*=0.001 for clonic/tonic seizure; *P*=0.045 for death) and NB001 effect (*P*=0.039 for wild running; *P*=0.001 for clonic/tonic seizure; *P*=0.146 for death). The numbers of animals are indicated in the table.

**Table 3:** Acute administration of rolipram has no significant effect on audiogenic seizures.

**Genotype and treatment**	***N*** **(number)**	**% Wild running**	**% Clonic/tonic seizure**	**% Death**
WT+vehicle	15	26.7	0	0
*Fmr1* KO+vehicle	13	62.5	46.2	15.4
WT+rolipram	16	25	0	0
*Fmr1* KO+rolipram	15	60	46.7	13.3

Audiogenic seizures were induced by sounds at the intensity of 120 dB. The percentage of different seizure-related phenotypes including wild running, clonic/tonic seizures, and death in WT and *Fmr1* KO receiving vehicle or rolipram (0.5 mg kg^−1^) is shown. χ^2^ test reveals significant genotype effect (*P*=0.007 for wild running; *P*=0.001 for clonic/tonic seizure; *P*=0.029 for death) but no rolipram effect (*P*=0.934 for wild running; *P*=0.743 for clonic/tonic seizure; *P*=0.502 for death). The numbers of animals are indicated in the table.
