# Supplementary material for: Enhanced expression of ADCY1 underlies aberrant neuronal signalling and behaviour in a syndromic autism model
Source: Nat Commun. 2017 Feb 20;8:14359. doi: 10.1038/ncomms14359 (PMC5321753; doi:10.1038/ncomms14359)
Supplement: Supplementary Information — Supplementary Figures and Supplementary Tables [file ncomms14359-s1.pdf]

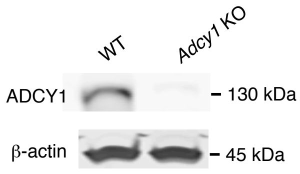

Supplementary Figure 1. Western blot of hippocampal lysates from WT and *Adcy1* KO mice demonstrates the specificity of the ADCY1 antibody.

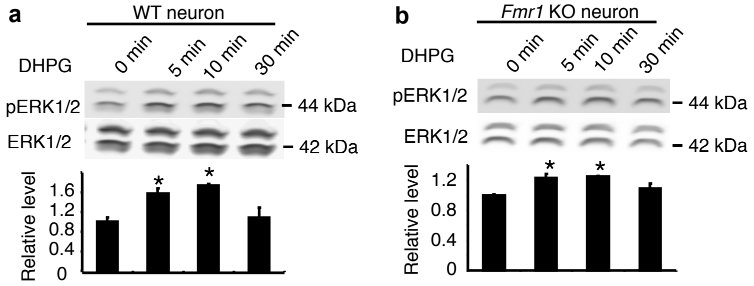

Supplementary Figure 2. The mGluR1/5-mediated intracellular signaling is intact in *Fmr1* KO neurons. The level of phosphorylated ERK1/2 (pERK1/2) and total ERK1/2 in WT hippocampal neurons **(a)** and *Fmr1* KO hippocampal neurons **(b)** following the treatment with mGluR1/5 agonist DHPG (100 μM) was determined by Western blot. Quantifications show the relative level of pERK1/2 normalized to total ERK1/2 (n=6 per group). One-way ANOVA and LSD test was used to determine p-value, * indicates $p<0.05$ between the 0 min group and the indicated group, one-way ANOVA followed by LSD post hoc analysis. Data are presented as mean ± SEM.

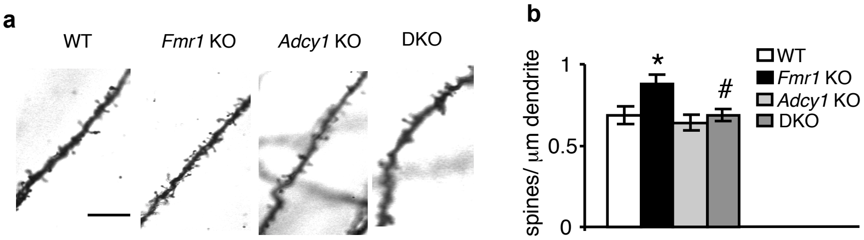

Supplementary Figure 3. Genetic deletion of *Adcy1* in *Fmr1 KO* mice rescues higher spine density in visual cortex. (a) Golgi staining of dendritic spines on the apical dendrites of pyramidal neurons in the visual cortex of WT, *Fmr1* KO, *Adcy1* KO, and *Fmr1/Adcy1* DKO mice (n = 4 for each genotype; length of the scale bar = 10 μm). (**b**) Quantification of total spine number. *: $p < 0.05$ between WT and indicated group, one-way ANOVA followed by LSD post hoc analysis. #: $p < 0.05$ between *Fmr1* KO and the indicated group, one-way ANOVA followed by LSD post hoc analysis. Data are presented as mean ± SEM.

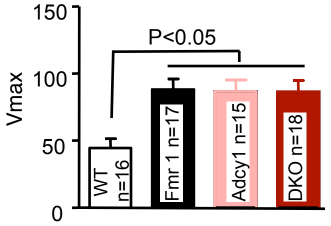

Supplementary Figure 4. Acoustic startle response in mice. Startle responses to 120 dB auditory stimulation were determined in four different mouse strains as indicated. Comparing to the WT mice, *Fmr1* KO, *Adcy1* KO, and *Fmr1/Adcy1* DKO animals showed higher responses. Data are presented as mean ± SEM.

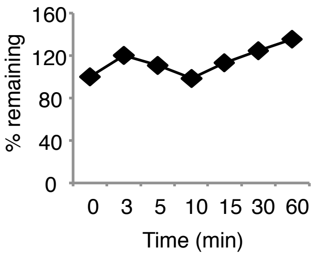

Supplementary Figure 5. The level of NB001 at different time points during incubation with liver microsomes.

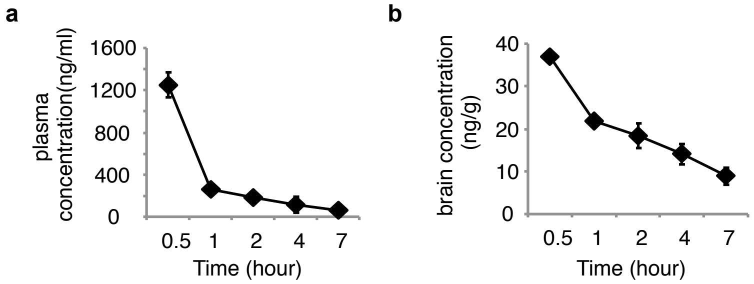

Supplementary Figure 6. Absorption and distribution of NB001 in plasma and brain. Samples prepared from plasma or brain were subjected to LC/MS/MS with Turbo-Ionspray^{TM} Interface in the positive ion-mode (Pharmacokinetics Core, University of Michigan). The concentration of NB001 in plasma (**a**) and brain (**b**) was determined at different time points following intraperitoneal injection.

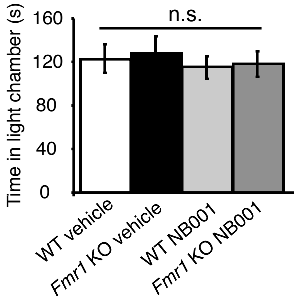

Supplementary Figure 7. Effect of NB001 on behavior in light/dark test. Time spent in the lit chamber during the light/dark test was recorded for WT and *Fmr1* KO mice injected with vehicle (WT, n = 11; *Fmr1* KO, n = 13) or 1 mg per kg NB001 (WT, n = 10; *Fmr1* KO, n = 15). n.s.: not significant. Data are presented as mean ± SEM.

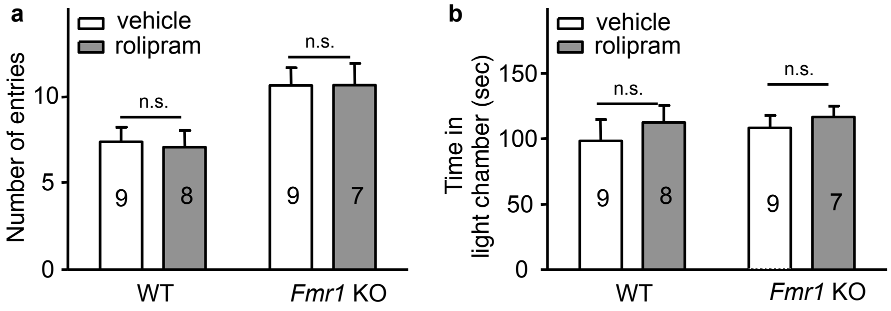

Supplementary Figure 8. Effects of acute low dose rolipram on behavior in the light/dark test. WT and *Fmr1* KO mice received i.p. injection of rolipram (0.03 mg per kg) or vehicle. Thirty minutes after injection, mice were examined by light/dark test as described in Figure 4. Regardless of treatment, *Fmr1* KO mice showed more transition between the lit and dark chamber than WT mice (genotype effect: $F_{(1,29)} = 8.0$, p=0.008) (**a**), and normal time in either lit or dark chamber (**b**). Rolipram does not cause significant behavioral changes in either WT or *Fmr1* KO mice (treatment effect: $F_{(1,29)} = 0$, p=1). n.s.: not significant. Data are presented as mean ± SEM.

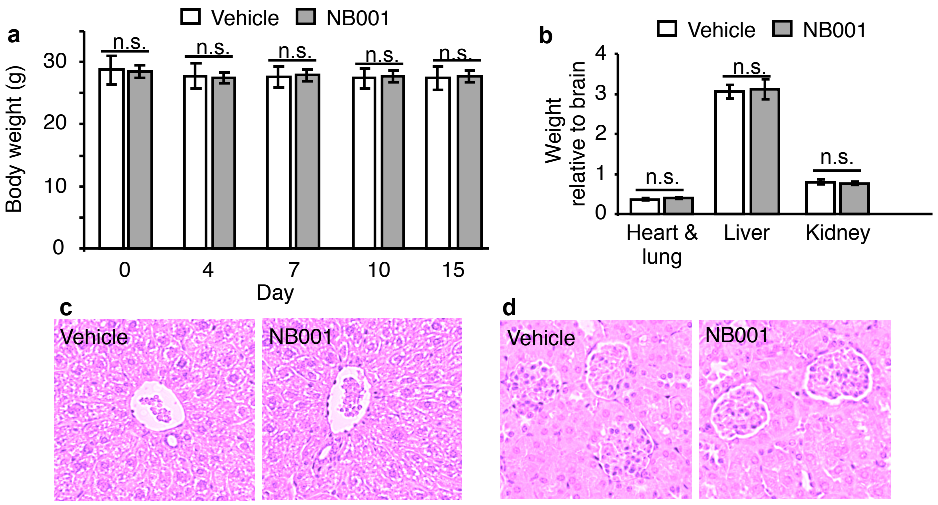

Supplementary Figure 9. There is no significant toxicity following two weeks of NB001 administration at high dose. NB001 (50 mg/kg) (n=5) or vehicle (n=3) was repeatedly administered twice per day for 14 days. **(a)** Body weight of mice at different days during the 2-week NB001 administration. **(b)** Weight of heart and lung, liver, and kidney normalized to brain weight was recorded at the end of 2-week NB001 administration. **(c, d)** H&E staining and histology of liver **(c)** and kidney **(d)** tissues after 14 days of NB001 administration. n.s.: not significant. Data are presented as mean ± SEM.

Supplementary Figure 10. Un-cropped Western blots.

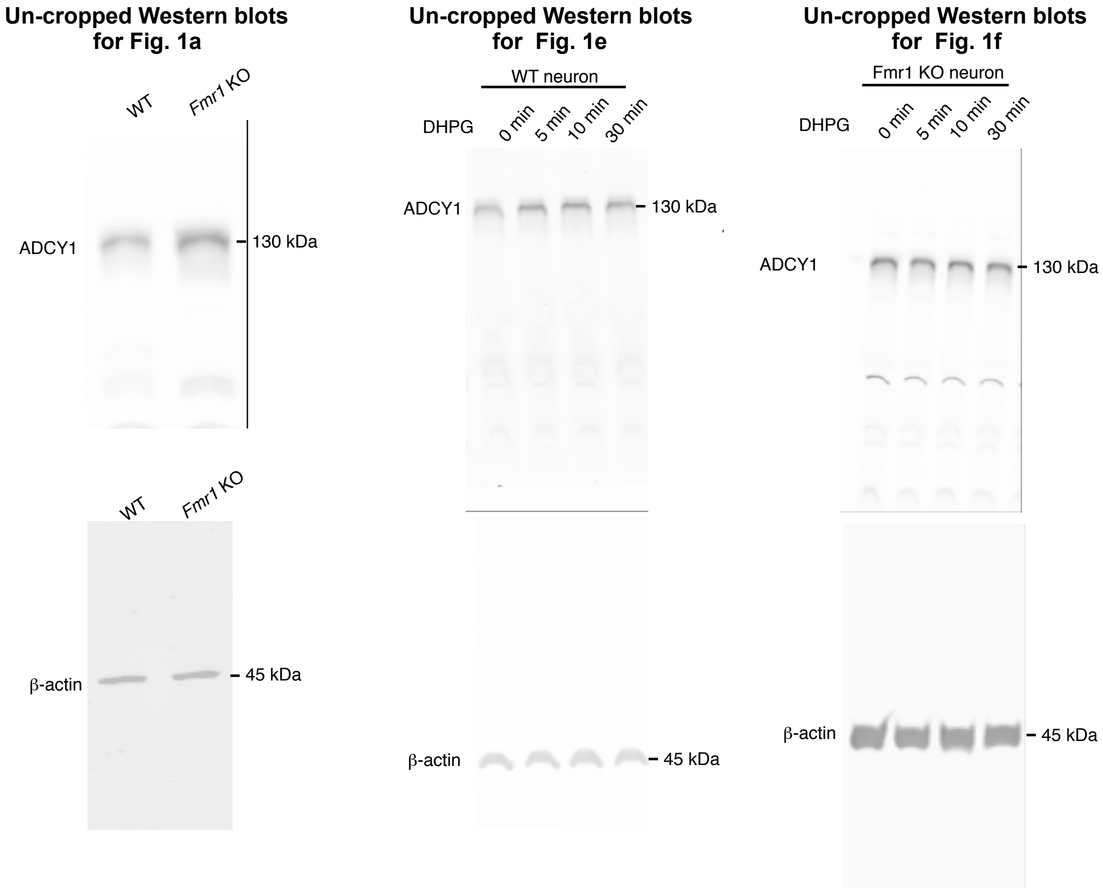

Un-cropped Western blots for Fig. 2a

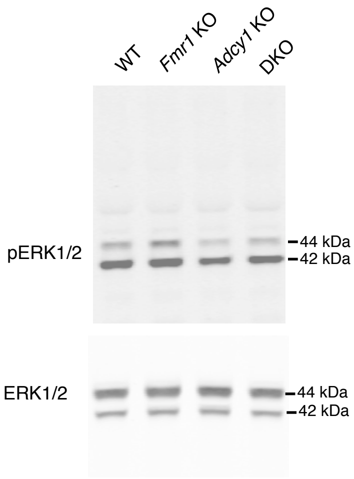

Un-cropped Western blots for Fig. 2b

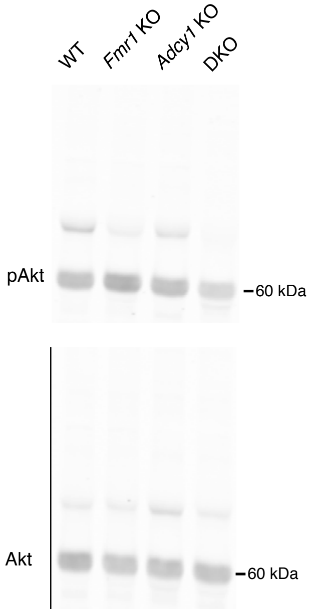

Un-cropped Western blots for Fig. 2c

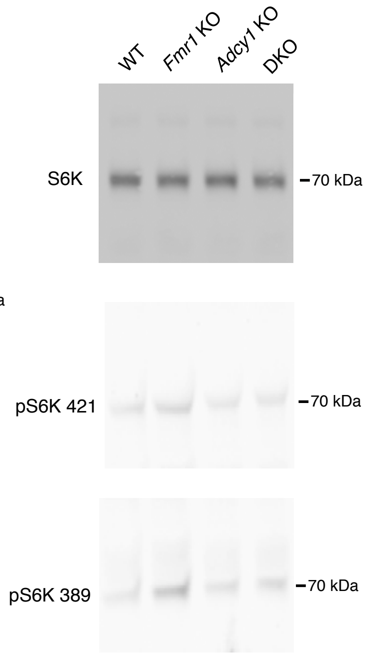

Un-cropped Western blots for Fig. 3a

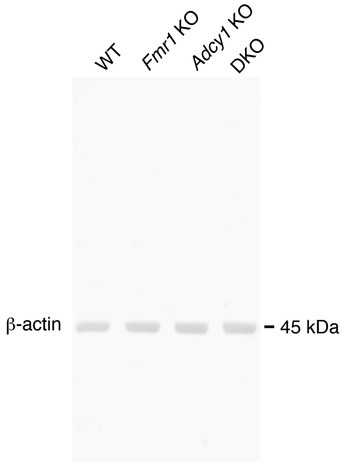

Un-cropped Western blots for Fig. 5b

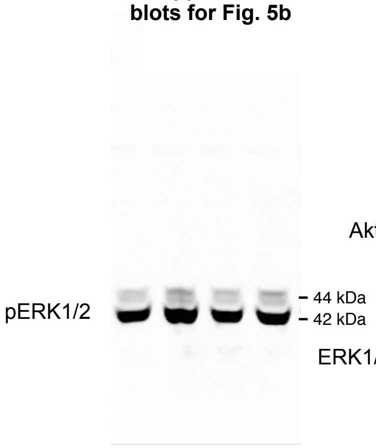

Un-cropped Western blots for Fig. 5b and 5d

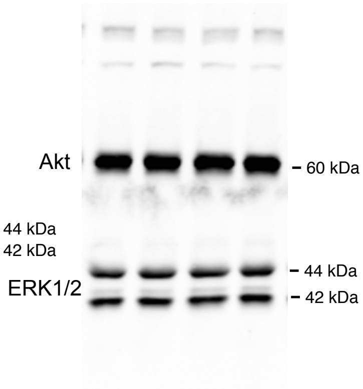

Anti Akt and anti ERK1/2 added at the same time

Un-cropped Western blots for Fig. 5d

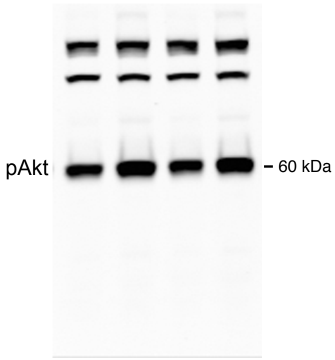

Un-cropped Western blots for Fig. 5c

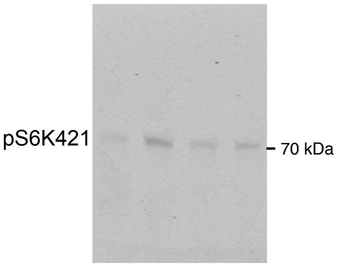

Un-cropped Western blots for Fig. 5c

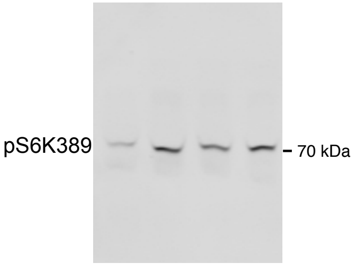

Un-cropped Western blots for Fig. 5c

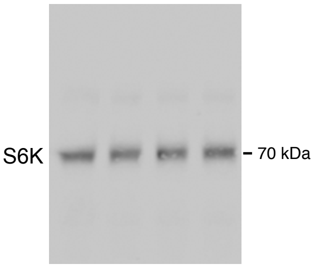

Un-cropped Western blots for Supplementary Fig. 1

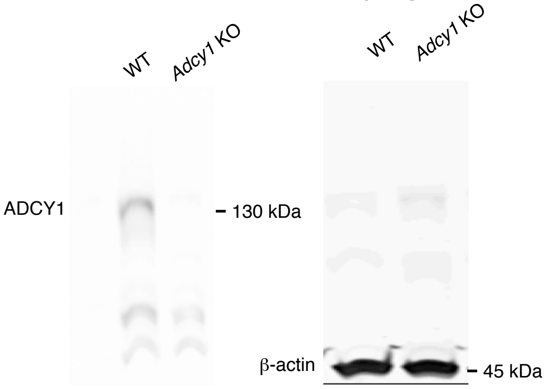

Un-cropped Western blots for Supplementary Fig. 2a

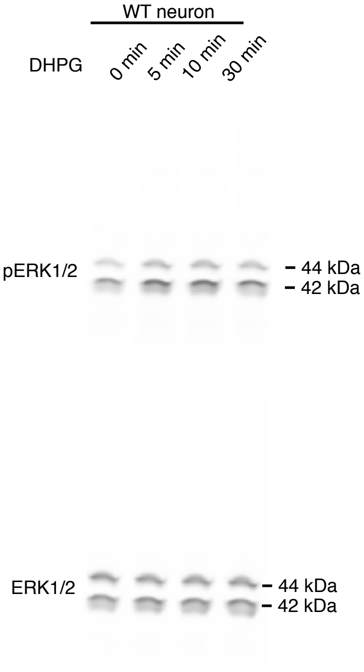

Un-cropped Western blots for Supplementary Fig. 2b

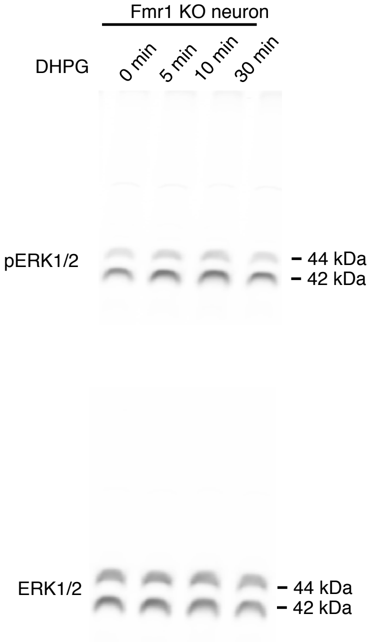

Supplementary Table 1. Effects of acute low dose rolipram on audiogenic seizures

Genotype and treatment	N number	% wild running	% clonic/tonic seizure	% death
WT + vehicle	15	13.3	0	0
Fmr1 KO + vehicle	13	46.2	30.8	15.4
WT + rolipram	15	13.3	0	0
Fmr1 KO + rolipram	14	50	35.7	14.3

Audiogenic seizures were induced by 120 dB auditory stimulation 30 min after rolipram (0.03 mg per kg) or vehicle (10% kolliphore) injection. The percentage of different seizure-related phenotypes including wild running, clonic/tonic seizures, and death is shown. Chi-square test reveals significant genotype effect ($p=0.004$ for wild running; $p=0.001$ for clonic/tonic seizure; $p=0.03$ for death) but no rolipram effect ($p=0.842$ for wild running; $p=0.745$ for clonic/tonic seizure; $p=0.936$ for death).

Supplementary Table 2. Long-term effects of NB001

	**Control**	**NB001**
Urea Nitrogen (mg/dL)	21.67 ±1.33	24.50 ±0.87
Sodium (mmol/L)	151.33 ±0.33	150.50 ±1.50
Potassium (mmol/L)	4.77 ±0.03	4.73 ±0.22
Chloride (mmol/L)	108.33 ±0.67	112.25 ±0.85
Total CO2 (mmol/L)	13.00 ±0.58	13.50 ±1.04
Anion Gap (mmol/L)	35.00 ±0.58	29.50 ±2.36
Na/K Ratio	31.67 ±0.33	32.00 ±1.47
Osmolarity (mOs/L)	321.00 ±1.00	321.00 ±4.00
Glucose (mg/dL)	183.50 ±15.50	216.50 ±8.50
Calcium (mg/dL)	8.60 ±0.00	8.83 ±0.20
Phosphorus (mg/dL)	7.37 ±0.18	6.50 ±0.24
Magnesium (mg/dL)	3.33 ±0.12	2.60 ±0.11
Iron (ug/dL)	107.00 ±44.00	115.50 ±4.50
Albumin (g/dL)	2.80 ±0.00	2.65 ±0.13
ALT (U/L)	22.67 ±1.45	18.50 ±0.96
AST (U/L)	40.33 ±1.86	32.50 ±1.55
ALP (U/L)	72.00 ±2.89	62.75 ±4.48
Amylase (U/L)	576.67 ±12.25	586.50 ±29.02
Chol (mg/dL)	95.67 ±4.81	100.00 ±5.96
Hemolysis Chem	Normal	Normal
Lipemia Chem	Normal	Normal
Icterus Chem	Normal	Normal

Blood test results following 14 days of NB001 administration. Vehicle or NB001 at 50 mg per kg (n=5) was given twice per day for 14 days, after which blood samples were collected. ALT: alanine aminotransferase, AST: aspartate aminotransferase, ALP: alkaline phosphatase. Data are presented as mean ± SEM.
